# Draft Genome Sequence of NRRL 5109, an Ex-Type Isolate of *Aspergillus neoellipticus*

**DOI:** 10.1128/MRA.01262-18

**Published:** 2018-12-06

**Authors:** Licui Li, Tom Hsiang, Qili Li, Long Wang, Zhihe Yu

**Affiliations:** a College of Life Sciences, Yangtze University, Jingzhou, Hubei, China; b School of Environmental Sciences, University of Guelph, Guelph, Ontario, Canada; c Institute of Plant Protection, Guangxi Academy of Agricultural Sciences, Nanning, Guangxi, China; d State Key Laboratory of Mycology, Institute of Microbiology, Chinese Academy of Sciences, Beijing, China; University of Maryland School of Medicine

## Abstract

We report here a draft genome sequence of an ex-type strain of Aspergillus neoellipticus, NRRL 5109, which was isolated from pus of a case of chronic emphysema. The final assembly consists of 160 scaffolds totaling 27.55 Mbp (G+C content, 49.96%) and 8,858 predicted genes.

## ANNOUNCEMENT

Aspergillus fumigatus *sensu lato* is known as a saprophytic fungus and an important opportunistic human pathogen distributed worldwide (1). Toxins produced by A. fumigatus endanger the health of humans and animals, resulting in huge economic losses (2). However, because of the superficial morphological resemblance among different species, expertise in Aspergillus taxonomy is required to recognize the taxa. There is controversy as to whether Aspergillus neoellipticus should be considered conspecific with A. fumigatus or treated as a separate species (3, 4). We attempted to quickly identify isolates of Aspergillus fumigatus and related taxa by designing five specific primers based on the sequences of gene RBPII and test primers on 34 isolates from 20 Aspergillus species and related taxa. The results showed that two pairs of primers, F2625/R3438 and F2255/R3044 (Y. Yu, L. Wang, Y. Wang, Z. Zeng, and Z. Yu, unpublished data), were able to specifically amplify DNA of 11 strains of A. fumigatus and A. neoellipticus but not that of 18 other species of Aspergillus tested. The Aspergillus neoellipticus isolate NRRL 5109^T^ was provided by the State Key Laboratory of Mycology (Institute of Microbiology, Chinese Academy of Sciences, Beijing, China) and had originated from pus from a case of chronic emphysema. Therefore, to gain a deeper understanding of this isolate, including pathogenicity and gene expression between closely related species using comparative genome analysis, especially to ascertain whether it should be regarded a separate species, the genome was sequenced.

The strain was cultured in potato-dextrose agar at 25°C for 5 days, and genomic DNA was recovered from fresh fungal cultures using a cetyltrimethylammonium bromide (CTAB) extraction method. The sequencing library was generated using the NEB Nextera DNA library prep kit (Illumina, Inc., San Diego, CA, USA). Whole-genome sequencing was performed by Génome Québec (Montreal, Canada) using a HiSeq 2000 platform, specifying 125-bp paired-end reads with a 300-bp insertion. Then, 21,147,236 paired-end reads totaling 6.2 Gb were generated. The quality of the reads was assessed with the program FastQC. The result showed consistent read quality that decreased steadily over the length of the reads, so the reads were used directly without trimming for *de novo* assembly. The genome was *de novo* assembled with the programs Velvet version 1.2.10 (5), ABySS version 1.9.0 (6), SOAP*denovo* version 2.04 plus GapCloser version 1.1.2 (7), MaSuRCA version 3.2.2 (8), and IDBA-UD version 1.1.3 (9), with odd-numbered k-mers between 31 and 91. Assembly quality and gene predictions using AUGUSTUS version 3.2.2 (10) based on gene models from Aspergillus nidulans were assessed by examining the *N*_50_ value and using Benchmarking Universal Single-Copy Orthologs (BUSCO) (11). The optimal assembly was obtained using ABySS at a k-mer of 55, with a total length of 27.55 Mb, consisting of 160 scaffolds, with the largest scaffold measuring 2,127,822 bp, and an *N*_50_ value of 886,472 bp. BUSCO based on OrthoDB version 9 data sets for Ascomycota identified 1,301 out of 1,315 complete genes in the assembly genome, containing 1,299 complete and single-copy genes. A BLASTP search using the 8,858 predicted genes against RefSeq and the two annotated genomes of A. fumigatus (GenBank accession numbers NC_007194 and DS499594) revealed significant matches (E value, 1E−5) for 8,789, 8,544, and 8,575 proteins, respectively. Compared with the sequences of A. fumigatus, this genome sequence could further help verify the species status of A. neoellipticus.

### Data availability.

This whole-genome shotgun project has been deposited at DDBJ/ENA/GenBank under the accession number QDFW00000000. Raw sequence reads (Illumina) have been deposited in the NCBI Sequence Read Archive under the BioProject number PRJNA449781.
